# Impact of urbanisation and altitude on the incidence of, and risk factors for, hypertension

**DOI:** 10.1136/heartjnl-2016-310347

**Published:** 2017-01-23

**Authors:** Antonio Bernabé-Ortiz, Rodrigo M Carrillo-Larco, Robert H Gilman, William Checkley, Liam Smeeth, J Jaime Miranda, CRONICAS Cohort Study Group

**Affiliations:** 1 CRONICAS Center of Excellence in Chronic Diseases, Universidad Peruana Cayetano Heredia, Lima, Peru; 2 Faculty of Epidemiology and Population Health, London School of Hygiene and Tropical Medicine, London, UK; 3 Department of International Health, Bloomberg School of Public Health, Johns Hopkins University, Baltimore, USA; 4 Área de Investigación y Desarrollo, Asociación Benéfica PRISMA, Lima, Peru; 5 Division of Pulmonary and Critical Care, School of Medicine, Johns Hopkins University, Baltimore, USA; 6 Department of Medicine, School of Medicine, Universidad Peruana Cayetano Heredia, Lima, Peru

## Abstract

**Background:**

Most of the data regarding the burden of hypertension in low-income and middle-income countries comes from cross-sectional surveys instead of longitudinal studies. We estimated the incidence of, and risk factors for, hypertension in four study sites with different degree of urbanisation and altitude.

**Methods:**

Data from the CRONICAS Cohort Study, conducted in urban, semiurban and rural areas in Peru, was used. An age-stratified and sex-stratified random sample of participants was taken from the most updated census available in each site. Hypertension was defined as systolic blood pressure ≥140 mm Hg, or diastolic blood pressure ≥90 mm Hg, or self-report physician diagnosis and current treatment. The exposures were study site and altitude as well as modifiable risk factors. Incidence, incidence rate ratios (IRRs), 95% CIs and population-attributable fractions (PAFs) were estimated using generalised linear models.

**Results:**

Information from 3237 participants, mean age 55.8 (SD±12.7) years, 48.4% males, was analysed. Overall baseline prevalence of hypertension was 19.7% (95% CI 18.4% to 21.1%). A total of 375 new cases of hypertension were recorded, including 5266 person-years of follow-up, with an incidence of 7.12 (95% CI 6.44 to 7.88) per 100 person-years. Individuals from semiurban site were at higher risk of hypertension compared with highly urbanised areas (IRR=1.76; 95% CI 1.39 to 2.23); however, those from high-altitude sites had a reduced risk (IRR=0.74; 95% CI 0.58 to 0.95). Obesity was the leading risk factor for hypertension with a great variation according to study site with PAF ranging from 12.5% to 42.4%.

**Conclusions:**

Our results suggest heterogeneity in the progression towards hypertension depending on urbanisation and site altitude.

## Introduction

Worldwide, the increasing number of cases of hypertension is mainly attributed to population growth, ageing and modifiable risk factors. The design of population-wide and individual strategies to prevent hypertension depends on the availability of reliable information.^1^ ^2^ However, most of the available data regarding the contribution of risk factors to the burden of hypertension in low/middle-income countries (LMICs) come from cross-sectional surveys instead of longitudinal prospective studies.

Urbanisation can negatively affect population health due to changes in diet and physical activity patterns,^3^ ^4^ with a subsequent increase on cardiovascular disease. In a previous cross-sectional study in Peru,^5^ a high degree of geographical variation was observed in the prevalence and distribution of risk factors for raised blood pressure. Peru, a middle-income country where more than half of all causes of deaths are due to non-communicable diseases,^6^ is characterised by the coexistence of urban, semiurban and rural settings, as well as high-altitude and low-altitude areas, providing thus a unique scenario to assess how urbanisation and altitude may impact, using a longitudinal approach, on the incidence of hypertension.

This study aimed to estimate the incidence of, and risk factors for, hypertension in four study sites with different degree of urbanisation and altitude in Peru. Besides, the overall and site-specific impact of modifiable risk factors on hypertension rates was assessed and population-attributable fractions (PAFs) were estimated.

## Methods

### Study design

Details about the CRONICAS Cohort Study have been published elsewhere.^7^ Briefly, this is an ongoing prospective cohort study conducted in four resource-limited settings in Peru featuring geographic differences: highly urbanised Lima and semiurban Tumbes, both at sea level, and urban and rural Puno at 3825 m above sea level. Information from the first two follow-up assessments, on average 15 and 30 months after baseline, was analysed.

### Study population

A random sample of participants stratified by age (35–44, 45–54, 55–64 and 65+ years) and sex was taken from the most updated census available in each study site. In each study site, 1000 individuals were enrolled; in Puno, however, 500 subjects were recruited in each urban and rural area. Individuals aged ≥35 years were eligible for the study, and only one person was recruited per household. Exclusion criteria included pregnant women, those who could not provide informed consent, were unable to respond to the questionnaires or were bedridden.^7^

### Variables definition

The main outcome of interest was the development of hypertension: systolic blood pressure (SBP) ≥140 mm Hg, or diastolic blood pressure (DBP) ≥90 mm Hg,^8^ or self-report physician diagnosis and currently receiving treatment for hypertension. Similarly, this definition was used to assess hypertension status at baseline (prevalence). Besides, SBP and DBP were also assessed as continuous variables. At baseline and follow-up evaluations, blood pressure was measured in triplicate after a 5 min resting period using an automatic monitor OMRON HEM-780 (OMRON, Tokyo, Japan) previously validated for adult population.^9^ Additionally, prehypertension was defined as an SBP from 120 to 139 mm Hg or DBP from 80 to 89 mm Hg without any treatment. Data about self-report physician diagnosis and current antihypertensive treatment were collected using standardised questionnaires applied by trained fieldworkers.

The exposure of interest was geographical variation assessed as urbanisation (highly urbanised Lima, urban Puno, semiurban Tumbes and rural Puno) and altitude (low-altitude vs high-altitude study sites). Modifiable risk factors assessed at baseline were also evaluated: daily smoking at least one cigarette per day (no/yes), self-reported; heavy alcohol drinking, defined as having had >1 night of alcohol intake in the previous month and having had drunk ≥6 drinks at the same time (no/yes); number of hours of TV watching per day (<2 and ≥2 hours per day), self-reported;^10^ ^11^ physical inactivity based on the leisure-time and transport-related domains of the International Physical Activity Questionnaire (IPAQ):^12^ leisure-time inactivity was defined as doing none or very little physical activity (ie, <600 MET-min/week) during leisure time, whereas transport-related inactivity was defined as not reporting walking or cycling trips (ie, a single walk or cycle trip for ≥10 min was considered to be classified as physically active);^13^ and fried food and high-sugar beverage consumption, both assessed separately with a frequency questionnaire with potential responses split into two categories (less than weekly vs weekly/daily). All these variables were assessed at baseline.

Additionally, other risk factors, based on anthropometric measurements, were assessed at baseline: body mass index (<25, 25–29.9 and ≥30 kg/m^2^);^14^ central obesity, based on waist circumference and categorised according to current guidelines for our population (<80 and ≥80 cm for females, and <90 and ≥90 cm for males);^15^ fasting total cholesterol (<200  and ≥200 mg/dL);^16^ metabolic syndrome, based on the joint scientific statement;^15^ and type 2 diabetes mellitus, defined as any of the following conditions: fasting glucose ≥126 mg/dL or self-report of physician diagnosis and currently receiving antihyperglycaemic drugs.^17^ Total cholesterol was measured on serum, whereas glucose was measured in plasma using an enzymatic colorimetric method (GOD-PAP; Modular P-E/Roche-Cobas, Grenzach-Whylen, Germany).

Other variables assessed at baseline and included in the analysis were sex (female or male); age (35–44, 45–54, 55–64 and ≥65 years); education (<7, 7–11 and ≥12 years) and socioeconomic status, assessed using a wealth index indicator based on assets and household facilities, and split in tertiles (low, middle and high).

### Procedures

Fieldwork activities have been published elsewhere.^7^ In brief, at baseline, fieldworkers visited households to contact potential participants and enrol them in the study. Subjects responded to a face-to-face questionnaire applied by trained community health workers using paper-based formats. After completing the questionnaire, an appointment for clinical evaluation was agreed to guarantee adequate fasting period (8–12 hours). A total of 13.5 mL of blood was drawn at baseline for cholesterol and glucose assessment. Standing height and abdominal circumference, in triplicate, was measured using standardised techniques. Weight was assessed using the TBF-300A body composition analyzer (TANITA Corporation, Tokyo, Japan).

### Statistical analysis

All the analyses were conducted using Stata V.13.0 for Windows (StataCorp, College Station, Texas, USA). Initially, a description of the study population was performed according to study site characteristics (urbanisation and altitude).

As incidence allows us to compare progression towards non-communicable conditions among populations, overall incidence rate of hypertension was estimated after excluding subjects with hypertension at baseline and reported per 100 person-years of follow-up. Incidence rates were also estimated according to population characteristics. The risk of developing hypertension according to study site and altitude was assessed using generalised linear models, assuming Poisson distribution, log-link function and including robust SEs. Crude and adjusted models were estimated controlling for different sociodemographic, behavioural and anthropometric confounders, reporting incidence rate ratios (IRRs) and their 95% CIs. Given the number of confounders and the potential correlation between them, the variance inflation factor was used to determine collinearity and exclude high correlated variables from the model if needed. Risk factors were also evaluated using the same strategy: adjusted models included age, sex, education level, socioeconomic status and study site as confounders. Overall and site-specific regression models were created to estimate IRRs, and PAFs were calculated using the *punaf* command in STATA.^18^

Differences in SBP and DBP variation, in mm Hg, during follow-up rounds were assessed for each exposure using repeated measures analysis of variance excluding participants with hypertension at baseline. In addition, random intercept models including two levels (assessments as level 1 units and subjects as level 2 clusters) were fitted. The crude model included the exposure of interest and the time of follow-up as categorical variable (baseline, 15 months and 30 months), whereas the adjusted models included, in addition, some potential confounders. Regression model results are presented as coefficients with their respective 95% CI.

### 

## Results

### Characteristics of the study population and baseline prevalence of hypertension

A total of 3237 participants were enrolled at baseline, mean age 55.8 (SD 12.7) years and 1565 (48.4%) were males. Characteristics and risk factor profiles of the study population, by site characteristics and hypertension status at baseline, are presented in Table 1 and online supplementary eTable 1, respectively. The prevalence of hypertension at baseline was 19.7% (95% CI 18.4% to 21.1%), whereas prehypertension was present in 24.9% (95% CI 23.4% to 26.4%).

**Table 1:** Population characteristics according to urbanisation and altitude at baseline

	Urbanisation				Altitude	
	Lima	Urban Puno	Tumbes	Rural Puno	Low altitude	High altitude
	(n=1052)	(n=574)	(n=581)	(n=1030)	(n=2082)	(n=1155)
Sociodemographics, n (%)						
Sex						
Female	546 (51.9%)	297 (51.7%)	311 (53.5%)	518 (50.3%)	1064 (51.1%)	608 (52.6%)
Age (years)						
<45	248 (23.6%)	139 (24.2%)	128 (22.1%)	260 (25.2%)	508 (24.4%)	267 (23.2%)
45–54	286 (27.2%)	139 (24.2%)	148 (25.6%)	251 (24.4%)	537 (25.8%)	287 (24.9%)
55–64	264 (25.1%)	147 (25.6%)	148 (25.6%)	261 (25.3%)	525 (25.2%)	295 (25.6%)
65+	254 (24.1%)	149 (26.0%)	155 (26.7%)	258 (25.1%)	512 (24.6%)	304 (26.4%)
Education level (years)						
< 7	454 (43.2%)	91 (15.9%)	373 (64.2%)	572 (55.6%)	1026 (49.3%)	464 (40.2%)
7–11	416 (39.6%)	156 (27.2%)	171 (29.4%)	312 (30.3%)	728 (35.0%)	327 (28.3%)
12+	181 (17.2%)	327 (56.9%)	37 (6.4%)	145 (14.1%)	326 (15.7%)	364 (31.5%)
Socioeconomic status						
Lowest tertile	127 (12.1%)	135 (23.5%)	419 (72.1%)	356 (34.6%)	483 (23.2%)	554 (48.0%)
Middle tertile	387 (36.8%)	156 (27.2%)	147 (25.3%)	401 (38.9%)	788 (37.9%)	303 (26.2%)
Highest tertile	538 (51.1%)	283 (49.3%)	15 (2.6%)	273 (26.5%)	811 (38.9%)	298 (25.8%)
Lifestyle behaviours, n (%)						
Daily smoking	34 (3.2%)	12 (2.1%)	1 (0.2%)	56 (5.4%)	90 (4.3%)	13 (1.1%)
Heavy alcohol drinking	57 (5.4%)	35 (6.1%)	16 (2.8%)	59 (5.7%)	116 (5.6%)	51 (4.4%)
TV watching (2+ hour/day)	509 (48.4%)	265 (46.2%)	83 (14.3%)	525 (51.0%)	1034 (49.7%)	348 (30.2%)
Leisure-time inactivity	974 (92.6%)	518 (90.2%)	558 (96.0%)	978 (95.0%)	1952 (93.8%)	1076 (93.2%)
Transport-related inactivity	75 (7.1%)	19 (3.3%)	12 (2.1%)	216 (21.0%)	291 (14.0%)	31 (2.7%)
Fried food consumption						
Weekly/daily	686 (65.2%)	427 (74.4%)	427 (73.5%)	684 (66.4%)	1370 (65.8%)	854 (73.9%)
High-sugar beverages consumption						
Weekly/daily	665 (63.2%)	301 (52.4%)	296 (51.0%)	409 (39.7%)	1074 (51.6%)	597 (51.7%)
*Measurements, n (%)*						
Body mass index (kg/m^2^)						
Normal (<25 kg/m^2^)	243 (23.2%)	135 (24.0%)	316 (54.5%)	255 (24.8%)	498 (24.0%)	451 (39.5%)
Overweight (≥25 and <30 kg/m^2^)	471 (45.0%)	278 (49.4%)	205 (35.3%)	450 (43.6%)	921 (44.4%)	483 (42.3%)
Obese (≥30 kg/m^2^)	332 (31.7%)	150 (26.6%)	59 (10.2%)	325 (31.6%)	657 (31.6%)	209 (18.3%)
Central obesity (IDF)	787 (75.2%)	428 (76.0%)	277 (47.8%)	845 (82.1%)	1632 (78.7%)	705 (61.7%)
Total cholesterol (≥200 mg/dL)	499 (48.4%)	250 (48.6%)	174 (32.3%)	539 (52.3%)	1038 (50.4%)	424 (40.3%)
Metabolic syndrome	505 (49.9%)	246 (47.9%)	150 (27.8%)	563 (54.7%)	1068 (51.8%)	396 (37.6%)
Hypertension	212 (20.2%)	79 (13.8%)	72 (12.4%)	277 (26.9%)	489 (23.5%)	151 (13.1%)
Type 2 diabetes mellitus	57 (5.5%)	37 (7.2%)	17 (3.2%)	106 (10.3%)	163 (7.9%)	54 (5.1%)

Results may not add due to missing values. IDF, International Diabetes Federation.

10.1136/annrheumdis-2016-210131.supp1 supplementary tables

### Incidence of hypertension and change of blood pressure levels

Of the total subjects evaluated at baseline, 196 (6.1%) were lost to follow-up, 38 (1.2%) died during follow-up and 641 (19.7%) were further excluded from incidence calculations because of their hypertension diagnosis at baseline. Among those lost to follow-up, 17.7% were from Lima, 43.7% were from urban Puno, 7.6% were from semiurban Tumbes and 31.0% were from rural Puno. Therefore, data from 2362 subjects were used in incidence estimations. Mean time of follow-up was 2.4 (SD 0.4) years, completing a total of 5266 person-years of follow-up. A total of 375 new cases of hypertension were found with an overall incidence of 7.12 (95% CI 6.44 to 7.88) per 100 person-years. Hypertension incidence, crude IRR and 95% CI according to population characteristics are shown in online supplementary eTable 2.

SBP mean at baseline (year 2010), first follow-up (at 15 months after baseline) and second follow-up (at 30 months after baseline) were 111.6 (SD 12.6), 111.9 (SD 14.3) and 114.0 (SD 15.9) mm Hg, respectively (p<0.001). Changes in DBP mean were from 70.7 (SD 8.5), 70.4 (SD 9.2) and 71.1 (SD 9.7) mm Hg in the same assessments (p=0.007).

### Study site, modifiable factors and the risk of hypertension

The incidence of hypertension was high in semiurban Tumbes compared with urbanised or rural study sites, and low in high-altitude areas (table 2). After controlling by potential confounders, individuals from semiurban Tumbes were at higher risk of developing hypertension compared with subjects from highly urbanised Lima (IRR=1.76; 95% CI 1.39 to 2.23), but there was no difference in the risk of hypertension in the other sites. In addition, individuals from high-altitude sites were at lower risk of hypertension (IRR=0.74; 95% CI 0.58 to 0.95).

**Table 2:** Association between study site characteristics and the risk of hypertension: crude and adjusted models

			Adjusted models		
	Incidence (95% CI)	Crude model	Model 1	Model 2	Model 3
	Per 100 person-years	IRR (95% CI)	IRR (95% CI)	IRR (95% CI)	IRR (95% CI)
Study site					
Lima	5.91 (4.92–7.10)	1 (reference)	1 (reference)	1 (reference)	1 (reference)
Urban Puno	5.73 (4.33–7.59)	0.98 (0.71–1.34)	1.01 (0.71–1.43)	1.01 (0.72–1.44)	1.02 (0.72–1.45)
Rural Puno	5.19 (3.76–7.16)	0.88 (0.62–1.26)	0.80 (0.55–1.16)	0.83 (0.57–1.21)	0.99 (0.68–1.45)
Tumbes	9.88 (8.52–11.5)	**1.67 (1.34–2.08)**	**1.75 (1.39–2.19)**	**1.79 (1.41–2.26)**	**1.76 (1.39–2.23)**
Site altitude					
Low	7.81 (6.96–8.77)	1 (reference)	1 (reference)	1 (reference)	1 (reference)
High	5.49 (4.44–6.78)	**0.71 (0.56–0.89)**	**0.65 (0.51–0.82)**	**0.67 (0.52–0.85)**	**0.74 (0.58–0.95)**

Bold estimates are statistically significant (p<0.05).

Model 1 was adjusted by sex, age, education level and socioeconomic status.

Model 2 was adjusted for sex, age, education level, socioeconomic status, daily smoking, heavy alcohol drinking, TV watching for 2+ hours per day, transport-related physical inactivity, fried food consumption and high-sugar beverage consumption.

Model 3 was adjusted for sex, age, education level, socioeconomic status, daily smoking, heavy alcohol drinking, TV watching for 2+ hours per day, transport-related physical inactivity, fried food consumption and high-sugar beverage consumption, body mass index and type 2 diabetes mellitus.

IRR, incidence rate ratios.

**Figure 1:**
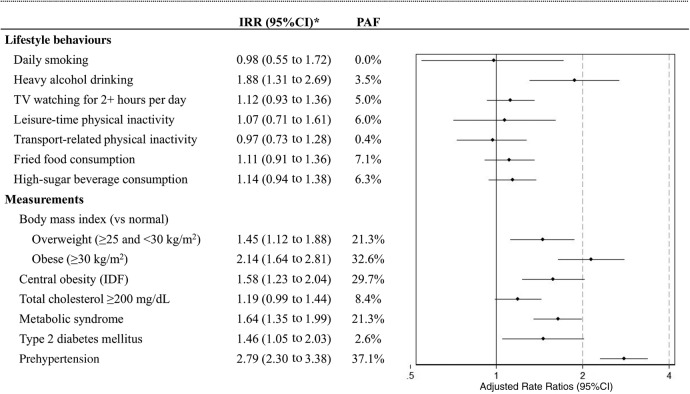
Modifiable factors and the risk of hypertension: adjusted models and population-attributable fractions (PAFs). *The model was adjusted by sex, age, education level, socioeconomic status and study site. IRR, incidence rate ratio.

In multivariable model, heavy alcohol drinking was the only modifiable risk factor associated with increased risk of hypertension (IRR=1.88; 95% CI 1.31 to 2.69). Variables related to obesity (body mass index, central obesity and metabolic syndrome) increased the risk of developing hypertension. PAF of obesity-related variables were >20% (figure 1); nevertheless, there was a wide variation between study sites: from 12.5% in urban Puno to 42.4% in rural Puno. Individuals with type 2 diabetes mellitus and prehypertension were also at greater risk of developing hypertension. However, the association between exposures of interest and risk of hypertension varied according to study site (see online supplementary eTable 3) and altitude (see online supplementary eTable 4).

### Determinants of SBP and DBP changes over time

In multivariable model, study site was a strong predictor of SBP and DBP changes over time (p<0.001); however, impact of urbanisation was not uniform ranging from −3.73 mm Hg in urban Puno to 4.76 mm Hg in semiurban Tumbes in the case of SBP compared with highly urbanised Lima (table 3). On the other hand, site altitude only modified SBP but not DBP over time. Among modifiable risk factors, heavy alcohol drinking (p<0.001) and high-sugar beverage consumption (p<0.001) increased both SBP and DBP during follow-up. All the anthropometric measurements and laboratory markers increased SBP and DBP across follow-up rounds.

**Table 3:** Systolic and diastolic blood pressure variation over time and associated factors: crude and adjusted linear mixed-effect models

	Systolic blood pressure (mm Hg)		Diastolic blood pressure (mm Hg)	
	Crude model	Adjusted model	Crude model	Adjusted model
	β (95% CI)	β (95% CI)	β (95% CI)	β (95% CI)
Sociodemographic variables*				
Study site (vs Lima)				
Urban Puno	**−3.35 (−4.65 to −2.06)**	**−3.61 (−4.85 to −2.36)**	0.67 (**−**0.18 to 1.52)	0.37 (**−**0.51 to 1.25)
Rural Puno	**−**0.99 (**−**2.25 to 0.26)	**−1.48 (−2.76** to **−0.21)**	**3.24 (2.45** to **4.03)**	**3.47 (2.59** to **4.36)**
Tumbes	**4.60 (3.39** to **5.80)**	**4.74 (3.63** to **5.85)**	**2.94 (2.20** to **3.68)**	**2.94 (2.20** to **3.69)**
Site altitude (vs low)				
High altitude	**−4.33 (−5.26** to **−3.40)**	**−5.03 (−5.91** to **−4.15)**	0.58 (**−**0.01 to 1.18)	0.40 (**−**0.22 to 1.01)
Lifestyle behaviours†				
Daily smoking	1.39 (**−**0.85 to 3.64)	**−**1.32 (**−**3.26 to 0.61)	1.05 (**−**0.43 to 2.53)	**−**0.56 (**−**2.01 to 0.90)
Heavy alcohol drinking	**5.02 (3.21** to **6.84)**	**3.05 (1.41** to **4.68)**	**3.53 (2.37** to **4.69)**	**1.91 (0.75** to **3.07)**
TV watching for 2+ hours per day	0.22 (**−**0.73 to 1.17)	0.07 (**−**0.79 to 0.93)	0.22 (**−**0.37 to 0.81)	0.27 (**−**0.31 to 0.85)
Leisure-time physical inactivity	**−1.89 (−3.58** to **−0.21)**	**−**1.44 (**−**3.05 to 0.17)	**−1.68 (−2.75** to **−0.61)**	**−**1.01 (**−**2.09 to 0.06)
Transport-related physical inactivity	**2.59 (0.75** to **4.43)**	**−**0.55 (**−**2.15 to 1.05)	0.08 (−0.93 to 1.09)	−0.38 (−1.38 to 0.62)
Fried food consumption	−0.42 (−1.46 to 0.62)	0.26 (−0.64 to 1.16)	0.49 (−0.15 to 1.14)	0.23 (−0.37 to 0.84)
High-sugar beverage consumption	0.83 (−0.11 to 1.78)	**1.06 (0.21** to **1.90)**	**1.20 (0.62** to **1.78)**	**1.05 (0.47** to **1.62)**
*Measurements*†				
Body mass index (vs normal)				
Overweight (≥25 and <30 kg/m^2^)	**1.83 (0.73** to **2.93)**	**3.45 (2.48** to **4.41)**	**1.68 (1.00** to **2.37)**	**2.49 (1.82** to **3.16)**
Obese (≥30 kg/m^2^)	**3.22 (1.93** to **4.50)**	**5.72 (4.55** to **6.88)**	**3.33 (2.54** to **4.12)**	**5.11 (4.31** to **5.91)**
Central obesity (IDF)	**1.58 (0.57** to **2.59)**	**3.96 (3.03** to **4.89)**	**1.44 (0.80** to **2.08)**	**3.08 (2.43** to **3.73)**
Total cholesterol ≥200 mg/dL	**2.82 (1.87** to **3.77)**	**2.82 (1.98** to **3.65)**	**1.61 (1.02** to **2.20)**	**1.91 (1.34** to **2.47)**
Metabolic syndrome	**4.54 (3.57** to **5.50)**	**4.79 (3.95** to **5.64)**	**2.74 (2.14** to **3.34)**	**3.32 (2.75** to **3.89)**
Type 2 diabetes mellitus	**4.07 (2.02** to **6.12)**	**2.81 (0.80** to **4.81)**	1.15 (−0.11 to 2.41)	**1.53 (0.26** to **2.81)**

Bold estimates are statistically significant (p<0.05).

*Each variable was adjusted by sex, age, education level, socioeconomic status and time of follow-up.

†Each variable was adjusted by sex, age, education level, socioeconomic status, study site and time of follow-up.

## Discussion

Individuals from semiurban sites were at higher risk compared with those from urbanised and rural sites. Moreover, the risk of hypertension was lower in high-altitude sites. Prehypertension and obesity were the leading risk factors for developing hypertension in all the study sites, but estimates, mainly in the case of obesity, varied depending on site's characteristics. Therefore, the results from this study can appropriately inform prevention and monitoring efforts including the identification of hot spot areas.

While the incidence of hypertension was similar between highly urbanised, urbanised and rural sites in the multivariable model, semiurban areas were at greater risk of hypertension introducing the need for further scrutiny to explain these results. Thus, according to PAF, leisure-time physical inactivity appears to be a problem in urban Puno and semiurban Tumbes (PAF >30%) but not in the other sites, whereas the effect of obesity was lower in urban Puno (PAF 12.5%) but high in the other sites (PAF ≥30%). The site in Tumbes, in the northern coast of Peru, is comprised by a group of communities with about 20 000 people spread over 80 km^2^, where the traditional agricultural landscape has become intermixed with rapidly growing urban sections. Thus, it seems that LMIC societal changes associated with growth and urbanisation are being telescoped into a much briefer time period relative to the lifespan of individuals.^19^

Individuals from high-altitude sites had reduced hypertension incidence rates compared with low-altitude sites. Individuals from high-altitude areas had greater PAF related to physical inactivity, fried food and high-sugar beverage consumption; PAF of obesity, however, was lower compared with low-altitude sites. These results support the fact that changes towards unhealthy diet and physical activity patterns are not homogeneous within country.^20^ ^21^ Previous reports, cross-sectional in nature, have shown divergent results. For example, a systematic review analysed different cross-sectional surveys from Tibet and suggested a positive correlation between altitude and the prevalence of hypertension.^22^ Conversely, lower rates of hypertension have been observed in individuals from high-altitude rural areas of Nepal.^23^ There is evidence that blood pressure does not vary in response to short-term hypoxic exposure;^24^ and increased blood pressure during altitude adaptation is explained by sympathetic nervous system activation.^25^ Permanent residence at high altitudes is associated with decrease in both SBP and DBP, perhaps secondary to chronic hypoxaemia. However, there is limited information about the physiological mechanisms of long-term exposure to high altitude and hypertension. Therefore, our study expands on previous findings by demonstrating the longitudinal impact of the environment and high altitude on the development of hypertension.

Among all the modifiable risk factors assessed in this study, only heavy alcohol drinking was associated with developing hypertension. Although alcohol has shown some beneficial effects on cardiovascular outcomes, heavy drinking increases the risk of hypertension as in previous studies,^26^ ^27^ yet its PAF was <5% in our population. Clinical variables such as high total cholesterol, metabolic syndrome and type 2 diabetes increased the risk of hypertension as previous studies have shown.^28^ ^29^

When using PAF, two different factors explained a high proportion of new cases of hypertension: obesity and prehypertension. Obesity was one of the leading risk factors for developing hypertension. The same pattern was observed in all the study sites and confirmed by the mixed-effect model. However, there was a great variation in the PAF estimates according to study site, thus confirming that the population-level effect of obesity on hypertension in each site is not uniform. Prehypertension was present in almost a quarter of the overall study population at baseline and was more frequent in semiurban sites (data not shown).^5^ Moreover, according to PAF, hypertension would be reduced by almost 40% in the study population if a reduction of blood pressure under 120/80 mm Hg were guaranteed, reinforcing the need for active screening of blood pressure levels or primary prevention strategies to avoid progression towards hypertension.

We report an overall hypertension incidence of 7 per 100 person-years. Our incidence estimates are similar to those reported for African-Americans in the USA, a population group that has been well characterised to have higher incidence rates relative to white subjects.^30^ Under these circumstances, our findings are worrying because they suggest our population could face the same disadvantages, in terms of cardiovascular health, as those faced by African-Americans. Our understanding of intervention strategies for non-communicable diseases in resource-poor settings is limited, if not absent, a disquieting fact given the trends of rise in SBP in many LMICs.^31^ The complex context of LMIC can provide a variety of scenarios that could identify new areas for innovation, relevant both at local and international levels, including Hispanic and Latino populations.^32^ ^33^

Our results lend support to actions where interventions designed to tackle hypertension could well be focused on obesity but through different pathways. For this, strategies should be implemented to promote mainly physical activity especially in the areas transitioning towards greater urbanisation, that is, urban and semiurban settings. In contrast, in rural areas, the focus of those interventions should be centred on securing patterns of healthy eating, mainly addressing reductions in the consumption of fried foods and sugar-added beverages.

This is a longitudinal study assessing the progression towards hypertension in different study sites with varying degree of urbanisation and altitude in an LMIC. Our estimates were calculated using a population-based study and standardised techniques. Nevertheless, some limitations should be considered. First, study site was used as a proxy of urbanisation process and altitude, and, for instance, some other unmeasured characteristics, that is, ethnicity, race, genetics, and so on, might have an impact on our results. For example, Quechua and Aymara groups are more frequent in highlands, especially in rural areas. Therefore, other prospective studies in other settings are required to corroborate our findings. Second, although we enrolled >3000 participants, statistical power might be an issue as well-recognised factors were not significant, perhaps because of short-term follow-up (30 months). However, as PAF assesses the contribution of a risk factor to a disease, they can provide a better understanding of the role of these factors in the involved populations. Third, some bias may arise as rejection rate at enrolment was different among study sites, being lower in semiurban Tumbes compared with the other sites (data not shown). Fourth, ambulatory blood pressure, considered the state-of-the-art way to assess hypertension, was not available, and instead only office blood pressure was used. Finally, although several variables were included in multivariable model, results may reflect the effect of unmeasured confounders. Diet and physical activity patterns were not completely assessed as only patterns of fried food and high-sugar beverage consumption and two domains of the IPAQ were included.

Our results are compatible with within-country heterogeneity in the developing of hypertension in resource-limited settings, lending support for focused and context-specific target interventions to reduce and tackle the burden of raised blood pressure for large populations. Obesity, assessed as body mass index and waist circumference, was the leading factor for developing hypertension in all the study sites. The development and implementation of interventions designed to address elevated blood pressure would benefit from accounting by geographical characteristics of low-income settings such as urbanisation and altitude.

**Key messages**

**What is already known on this subject?**

- Most of the data regarding the burden of hypertension in low-income and middle-income countries arise from cross-sectional surveys rather than longitudinal studies. Urbanisation can negatively affect the health of populations through changes in the profiles of diet and physical activity, with a subsequent increase on cardiovascular disease.

**What might this study add?**

- Our results show the effect of geographical variation, namely urbanisation and altitude, on hypertension rates. Obesity and prehypertension were the leading factors for developing hypertension in all the study sites, but estimates, mainly in the case of obesity, varied depending on site's characteristics.

**How might this impact on clinical practice?**

- The assessment of elevated blood pressure and hypertension in low-income settings requires embracing geographical and contextual factors. Obesity and prehypertension were the leading factors increasing the risk for hypertension, yet population-attributable fractions were not uniform across study sites.
