# Supplementary material for: Impact of urbanisation and altitude on the incidence of, and risk factors for, hypertension
Source: Heart. 2017 Jan 23;103(11):827–33. doi: 10.1136/heartjnl-2016-310347 (PMC5529980; doi:10.1136/heartjnl-2016-310347)
Supplement: supplementary tables [file heartjnl-2016-310347supp001.pdf]

ONLINE SUPPLEMENT

E-Table 1: Baseline population characteristics according to the presence of hypertension

	**Without hypertension**	**With hypertension**	
	**(n = 2,597)**	**(n = 640)**	**p-value**
**Socio-demographics, n (%)**			
Sex			0.96
Female	1,342 (51.6%)	330 (51.6%)	
Age			<0.001
< 45 years	727 (28.0%)	48 (7.5%)	
45 – 54 years	721 (27.8%)	104 (16.2%)	
55 – 64 years	641 (24.7%)	179 (28.0%)	
65 + years	508 (19.5%)	309 (48.3%)	
Education level			<0.001
< 7 years	1,100 (42.4%)	390 (60.9%)	
7 – 11 years	898 (34.6%)	157 (24.5%)	
12+ years	597 (23.0%)	93 (14.5%)	
Socioeconomic status			0.27
Lowest tertile	817 (31.4%)	220 (34.4%)	
Middle tertile	890 (34.3%)	201 (31.4%)	
Highest tertile	890 (34.3%)	219 (34.2%)	
Study site			<0.001
Lima	840 (32.3%)	212 (33.1%)	
Urban Puno	495 (19.1%)	79 (12.3%)	
Rural Puno	509 (19.6%)	72 (11.3%)	
Tumbes	753 (29.0%)	277 (43.3%)	
Site altitude			<0.001
High	1,004 (38.7%)	151 (23.6%)	
**Lifestyles behaviors, n (%)**			
Daily smoking			0.03
Yes	74 (2.9%)	29 (4.5%)	
Heavy alcohol drinking			0.69
Yes	136 (5.2%)	31 (4.8%)	
TV watching for 2+ hours per day			0.14
Yes	1,093 (42.1%)	289 (45.3%)	

Leisure-time physical inactivity			0.04
Yes	2,418 (93.1%)	610 (95.3%)	
Transport-related physical inactivity			<0.001
Yes	208 (8.0%)	114 (17.8%)	
Fried food consumption			<0.001
Weekly/daily	1,828 (70.4%)	396 (61.9%)	
High-sugar beverages consumption			<0.001
Weekly/daily	1,402 (54.0%)	269 (42.0%)	
**Measurements, n (%)**			
Body mass index			<0.001
Normal (<25 kg/m2)	803 (30.9%)	146 (23.5%)	
Overweight (≥25 and <30 kg/m2)	1,159 (44.6%)	245 (39.4%)	
Obese (≥30 kg/m2)	635 (24.5%)	231 (37.1%)	
Waist circumference (IDF)			<0.001
Central obesity	1,831 (70.5%)	506 (81.4%)	
Total cholesterol			<0.001
≥200 mg/dL	1,127 (44.9%)	335 (55.3%)	
Metabolic syndrome			< 0.001
Yes	1,006 (40.1%)	458 (75.6%)	
Type 2 diabetes mellitus			< 0.001
Yes	130 (5.2%)	87 (14.3%)	

Results may not add due to missing values. P-values were calculated using Chi-squared test.

E-Table 2: Incidence of hypertension according to population characteristics

	Incidence (95%CI)	Bivariable model
	**per 100 person-years**	**IRR (95%CI)**
**Socio-demographics**		
Sex		
Female	7.08 (6.15 - 8.15)	1 (reference)
Male	7.17 (6.20 – 8.29)	1.01 (0.84 – 1.22)
Age		
35 - 44 years	3.60 (2.76 – 4.69)	1 (reference)
45 – 54 years	4.98 (3.97 – 6.24)	1.38 (0.99 – 1.93)
55 – 64 years	8.84 (7.37 – 10.6)	2.45 (1.80 – 3.32)
65 + years	14. 1 (11.9 – 16.7)	3.91 (2.91 – 5.25)
Education level		
< 7 years	8.95 (7.77 – 10.3)	1 (reference)
7 – 11 years	6.70 (5.63 – 7.98)	0.75 (0.61 – 0.92)
12+ years	4.57 (3.52 – 5.94)	0.51 (0.39 – 0.68)
Socioeconomic status		
Lowest tertile	8.14 (6.80 – 9.73)	1 (reference)
Middle tertile	6.55 (5.49 – 7.82)	0.80 (0.64 – 1.01)
Highest tertile	6.89 (5.81 – 8.18)	0.85 (0.67 – 1.07)
**Lifestyles behaviors**		
Daily smoking		
No	7.13 (6.44 – 7.90)	1 (reference)
Yes	6.75 (3.74 – 12.2)	0.94 (0.53 – 1.67)
Heavy alcohol drinking		
No	6.96 (6.27 – 7.73)	1 (reference)
Yes	9.92 (6.85 – 14.4)	1.42 (0.99 – 2.04)
TV watching		
<2 hours per day	6.96 (6.07 – 7.99)	1 (reference)
2+ hours per day	7.32 (6.30 – 8.50)	1.05 (0.87 – 1.27)
Leisure-time physical inactivity		
No	5.69 (3.71 – 8.73)	1 (reference)
Yes	7.23 (6.51 – 8.02)	1.27 (0.84 – 1.92)

Transport-related physical inactivity		
No	6.94 (6.23 – 7.73)	1 (reference)
Yes	9.03 (6.65 – 12.3)	1.30 (0.97 – 1.74)
Fried food consumption		
< Weekly or daily	7.19 (5.98 – 8.63)	1 (reference)
Weekly or daily	7.09 (6.28 – 8.01)	0.99 (0.81 – 1.21)
High-sugar beverage consumption		
< Weekly or daily	7.38 (6.38 – 8.55)	1 (reference)
Weekly or daily	6.89 (5.99 – 7.93)	0.93 (0.77 – 1.13)
**Measurements**		
Body mass index		
Normal (<25 kg/m2)	5.32 (4.27 – 6.62)	1 (reference)
Overweight (≥25 and <30 kg/m2)	6.60 (5.65 – 7.71)	1.24 (0.96 – 1.60)
Obese (≥30 kg/m2)	10.1 (8.51 – 11. 9)	1.89 (1.46 – 2.45)
Waist circumference (IDF)		
No central obesity	5.02 (3.99 – 6.33)	1 (reference)
Central obesity	7.91 (7.06 – 8.85)	1.57 (1.23 – 2.00)
Total cholesterol		
<200 mg/dL	6.25 (5.39 – 7.23)	1 (reference)
≥200 mg/dL	8.15 (7.08 – 9.38)	1.30 (1.08 – 1.58)
Metabolic syndrome		
No	5.49 (4.72 – 6.39)	1 (reference)
Yes	9.42 (8.22 – 10.8)	1.71 (1.42 – 2.07)
Type 2 diabetes		
No	6.86 (6.17 – 7.63)	1 (reference)
Yes	12.0 (8.42 – 17.0)	1.74 (1.26 – 2.41)
Pre-hypertension		
No	4.31 (3.69 – 5.03)	1 (reference)
Yes	13.6 (11.9 – 15.5)	3.15 (2.61 – 3.81)

E-Table 3: Modifiable factors and the risk of hypertension according to study setting: adjusted models and population attributable fractions (PAF)

	Lima		Urban Puno		Rural Puno		Tumbes	
	**IRR***	**PAF**	**IRR***	**PAF**	**IRR***	**PAF**	**IRR***	**PAF**
**Lifestyles behaviors**								
Daily smoking	0.71	-0.7%	1.78	0.9%	--	--	1.08	0.3%
Heavy alcohol drinking	1.48	2.0%	**3.27**	8.5%	--	--	**2.19**	4.7%
TV watching for 2+ hours per day	0.94	-2.7%	**1.88**	25.8%	**0.72**	-3.2%	1.11	5.2%
Leisure-time physical inactivity	0.73	-32.9%	1.60	34.6%	**--**	--	1.49	31.9%
Transport-related physical inactivity	0.50	-2.6%	--	--	**--**	--	1.09	1.7%
Fried food consumption	1.03	1.7%	2.01	42.0%	1.39	21.4%	1.01	0.7%
High-sugar beverage consumption	0.88	-8.0%	1.46	18.8%	1.28	11.3%	1.23	8.4%
**Measurements**								
Body mass index (vs. normal)								
Overweight (≥25 and <30 kg/m^2)	1.21	14.7%	0.78	-12.7%	**3.19**	34.5%	**1.53**	26.7%
Obese (≥30 kg/m^2)	**2.19**	29.7%	1.03	12.5%	**4.57**	42.4%	**2.03**	35.1%
Central obesity (IDF)	**1.86**	38.5%	0.99	-0.6%	**2.48**	33.8%	1.43	25.9%
High cholesterol	1.15	6.5%	1.25	10.9%	1.84	17.3%	1.14	7.1%
Metabolic syndrome	**1.77**	24.8%	0.73	-14.1%	2.24	22.5%	**1.78**	26.6%
Type 2 diabetes mellitus	**2.11**	5.6%	0.73	-2.3%	1.68	3.3%	1.13	0.9%
Pre-hypertension	**3.30**	41.0%	**3.16**	30.7%	**2.37**	34.4%	**2.56**	36.9%

(--) = not calculable. Bold estimates are significant ($p < 0.05$).
* Model adjusted for sex, age, education level, and socioeconomic status.

E-Table 4: Modifiable factors and the risk of hypertension according to setting altitude: adjusted models and population attributable fractions (PAF)

	Low altitude		High altitude	
	**IRR***	**PAF**	**IRR***	**PAF**
**Lifestyles behaviors**				
Daily smoking	1.04	0.1%	1.68	0.5%
Heavy alcohol drinking	**1.86**	3.5%	2.18	3.8%
TV watching for 2+ hours per day	1.08	3.6%	**1.59**	12.9%
Leisure-time physical inactivity	1.01	1.3%	1.85	43.8%
Transport-related physical inactivity	1.20	2.4%	--	--
Fried food consumption	1.03	1.7%	1.63	31.2%
High-sugar beverage consumption	0.97	-1.3%	1.38	15.4%
**Measurements**				
Body mass index (vs. normal)				
Overweight (≥25 and <30 kg/m^2)	**1.47**	22.5%	1.48	18.6%
Obese (≥30 kg/m^2)	**2.10**	36.3%	**2.05**	22.3%
Central obesity (IDF)	**1.71**	35.1%	1.45	20.9%
High cholesterol	1.19	8.7%	1.42	14.1%
Metabolic syndrome	**1.85**	27.2%	1.17	5.7%
Type 2 diabetes mellitus	**1.54**	3.1%	1.17	1.0%
Pre-hypertension	**2.91**	39.3%	**2.79**	32.9%

(--) = not calculable. Bold estimates are significant ($p < 0.05$).
* Model adjusted for sex, age, education level, and socioeconomic status.
